# Practice makes perfect, especially when doing what we like

**DOI:** 10.3758/s13414-025-03031-8

**Published:** 2025-02-24

**Authors:** Irene Reppa, Siné McDougall

**Affiliations:** 1 School of Psychology, Swansea University, Swansea, SA2 8PP UK; 2 Department of Psychology, Bournemouth University, Bournemouth, UK

**Keywords:** Attention, Aesthetic appeal, Practice effects, Visual search

## Abstract

Previous research has found that aesthetic appeal can facilitate visual search performance. One avenue of enquiry is that appealing icons are processed better than unappealing icons. If appealing stimuli are better processed, then it may be expected that they will benefit from practice more than their unappealing counterparts. In the current study (*N* = 100) we examined the effect of stimulus appeal on visual search performance. Half of the participants searched for appealing icons first, followed by unappealing icons, and the order was reversed for the other half. First, visual search performance benefited from stimulus appeal, and specifically the interaction of stimulus appeal and complexity – visual stimulus appeal led to better search performance but only for stimuli that were visually complex, with no effect of appeal for visually simple stimuli. Second, task experience benefited appealing icons more than unappealing icons. These results extend current knowledge of the status of visual aesthetic appeal on performance. They provide new evidence that appealing stimuli benefit from practice and are easier to learn compared to their unappealing counterparts.

**Supplementary information:**

The online version contains supplementary material available at 10.3758/s13414-025-03031-8.

## Introduction

Over the last 20 years research in psychology and human-computer interaction has shown that aesthetic appeal influences our behaviour across a range of everyday life activities and it has a significant socio-economic impact. The pursuit of aesthetic experiences means we go to art galleries and buy beautiful things for our homes. While aesthetic appeal can refer to our thinking and deliberations with respect to works of art in a gallery, a much more common experience of appeal arises from the mild experiences of liking aroused by the impression stimuli make on our senses (from the Greek word *aesthesis* meaning *sensing*). We determine an object’s appeal fast, within 50 ms (e.g., Jacobsen et al., 2004; Lindgaard et al., 2006). It is one of the first judgements we make about an object.

### The role of appeal in visual search

Stimulus appeal also enhances performance in different time-critical tasks including visual search (e.g., Moshagen et al., 2009; Reppa & McDougall, 2015; Reppa et al., 2021; Sonderegger & Sauer, 2010; for review, see Thielsch et al., 2019). In the current study we sought to garner converging evidence for the effect of appeal on visual search, using a different design and comparing the effects obtained both when previous stimuli were used (see Experiment 1) and new stimuli in Experiment 2. Icons were used as stimuli because they are pervasive in interfaces in everyday life, often where responses are time-critical and where locating an appropriate icon via visual search of the interface is a large task component. The use of novel contemporary stimuli in Experiment 2 was important because icon design has changed considerably over time (for discussion of the need for large representative sets of stimuli and changes in icon design, see Brysbaert, 2007; Clark, 1973; Collaud et al., 2022).

The effects of icon characteristics are well documented and measured allowing appropriate experimental manipulation and control (see Reppa & McDougall, 2015, p. 1244, for further details). As in previous research, both the visual appeal and visual complexity of the icons was varied while holding their meaningfulness constant (see *Methods* section for details). Given previous findings, it was expected that appeal would have a significant beneficial effect on search performance with a greater benefit for simple over complex stimuli (e.g., Reppa & McDougall, 2015, 2022; Reppa et al., 2021). Similarly, *efficiency* of search was expected to be better for appealing and poorer for unappealing icons. This would be evident from a lower gradient on search response time curves as set size increased from three to 12 items (e.g., Reppa & McDougall, 2022).

### Task experience and task switching in visual search

**Fig. 1:**
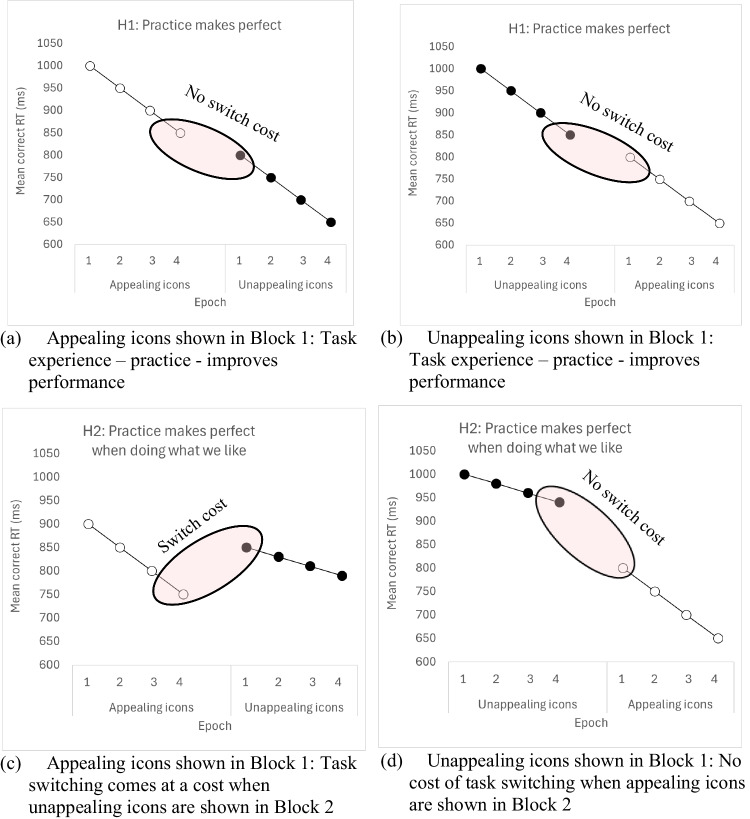
Prediction graphs for the interaction between task experience and appeal on visual search performance. If practice with the visual search task improves performance regardless of the appeal of the stimulus (H1 panel, top), then we might expect that search times will improve from block 1 to block 2, regardless of the appeal of the icons. However, if practice with the search task interacts with stimulus appeal, then we might expect that following practice with the search task in block 1, search times are even faster in block 2, but only when block 2 involves searching for appealing icons (H2 panel, bottom). No benefit of task experience is predicted when participants gain search experience with appealing icons in block 1, and block 2 involves searching unappealing icons (H2 panel, bottom). (a) Appealing icons shown in Block 1: Task experience – practice - improves performance, (b) Unappealing icons shown in Block 1: Task experience – practice - improves performance, (c) Appealing icons shown in Block 1: Task switching comes at a cost when unappealing icons are shown in Block 2, (d) Unappealing icons shown in Block 1: No cost of task switching when appealing icons are shown in Block 2

A novel combination of standard visual search and task-switching paradigms made it possible to examine whether the effects of stimulus appeal result from simply gaining more experience with the icon set or are the result of the intrinsic appeal of the icon stimuli. In both experiments, participants were presented with two blocks of visual search trials in which either only appealing or unappealing icons were presented as targets. Order of presentation was counterbalanced across participants so that half saw appealing targets first while the other half saw unappealing icons first. Our first hypothesis was that, if search times depend primarily on practice effects, then search times would decrease in the second block of search trials irrespective of the appeal of the targets (see Fig. 1a and c). This hypothesis was plausible because, like a multitude of other tasks, visual search has been shown to improve with task experience or practice (e.g., Clark et al., 2015; Hout & Goldinger, 2010; Sireteanu & Rettenbach, 1995; Sigman & Gilbert, 2000).

A second, alternative, hypothesis relates to the ‘task switching’ involved between blocks when participants needed to switch from search for one type of icon to another. The notion of task switching in this instance depends on whether or not presentation of appealing and unappealing icons has an effect on visual search *in and of itself* (i.e., dependent on the nature of the stimulus), which is separate from simply gaining experience with the search task. If this is the case, then the expectation would be that changing the appeal of the icons is effectively creating a new attentional set for participants to adapt to.

In task-switching studies (e.g., Dombrowe et al., 2011; Monsell, 2003; Rogers & Monsell, 1995), where an appropriate response is produced in response to a stimulus (e.g., pressing the ‘up’ arrow if a coloured square appears at the top of the screen, and the ‘down’ arrow when it appears at the bottom of the screen), a key finding is that switching a task or an attentional set (switch to pressing the ‘up’ arrow when the coloured square is yellow and the ‘down’ arrow when it is blue) often comes with a cost in response accuracy and efficiency. That is then compared to performance when the tasks remain the same, which is associated with superior performance as a result of maintaining the same attentional set (for review, see Monsell, 2003).

Our hypothesis was that appeal is an exogenous stimulus attribute that would influence attentional set and that the change from one set of stimuli to the other would differ depending on the appeal of the icons when switched in block 2. Specifically, following practice with the search task in block 1, search times are even faster in block 2, but only when block 2 involves searching for appealing icons (Fig. 1d). No benefit of task experience is predicted when participants gain search experience with appealing icons in block 1, and block 2 involves searching unappealing icons – that is, there will be a cost when switching to unappealing

icons (Fig. 1c). To investigate how practice interacts with stimulus appeal, we used a method commonly employed in the literature that examines search performance over smaller sets of trials. Following Endo and Takeda (2005) and Hout and Goldinger (2010), response times (RTs) were divided into *epochs*, with each epoch comprising 25% of each block’s trials. A main effect of Epoch would indicate that RTs significantly decreased as the trials progressed within each block.

### The effect of appeal on mood and arousal

Recently, Reppa et al. (2021) presented evidence to support the notion that it is the *motivating* nature of appealing stimuli that facilitates performance. At the start of the experimental trials, participants were induced into a positive or a negative mood. Participants in a positive mood were biased toward detecting aesthetically pleasing icons, detecting them more quickly as a result. Initially participants induced into a negative mood showed no advantage for appealing icons but, as experience with appealing icons increased, participants in the negative mood state gradually began to show performance benefits when presented with appealing icons. These findings suggested that the benefit for appealing over unappealing icons may be the result of the reinforcing power of aesthetically pleasing stimuli, which builds over time (e.g., Mackintosh, 1975; see also Han et al., 2020, for similar findings using attractive and unattractive faces). For those in an initially negative mood, this reinforcement took more time to have an effect than for this initially induced into a positive mood state. In Experiments 1 and 2 the motivational effect of appeal on performance was examined further. The experimental design, which involved the combination of gaining experience with the visual search task and task switching, made it possible to examine whether or not gradual effects observed for those in an initial negative mood were due to simply gaining familiarity with the visual search task or related more specifically to the appeal (or lack of appeal) of the stimuli being presented. To this end, participants’ mood was measured prior to the visual task to create a baseline measure and then subsequently after presentation of blocks 1 and 2 of visual search trials.

To summarise, the experiments reported here used a visual search paradigm to examine the role of appeal in performance further. This study had the following aims:

- To replicate previous findings using previously existing and novel, up-to-date, stimulus sets.
- To examine whether the effects of stimulus appeal on search performance result from simply gaining experience with the visual search task or from the intrinsic appeal of the stimuli themselves. Separation of these effects was possible using a combination of a standard visual search task and a task switching design.
- To examine how participants’ initial mood, either positive or negative, may interact with the appeal of the icon stimuli in determining visual search performance (i.e., depends on the intrinsic appeal of the stimuli or depends on gaining experience with the icons, irrespective of their appeal).

## Experiment 1: The effects of icon appeal on visual search using the original icon set

### Method

#### Participants

Fifty participants were recruited from the student population at Swansea University via the participant pool (32 female, 18 male, age 18–23 years), with reported normal or corrected-to-normal vision. They received four participant pool credits for approximately 50 min of their time The number of participants in visual search experiments manipulating variables within-participants varies from around 10 to 25. G-Power analysis for a mixed ANOVA using a rejection criterion of *p* < .05, 90% power, and a large effect size based on previous data (*d* = .40), suggested a sample size of 23 was needed per group. Extra participants were recruited in case of attrition with 25 participants in the appealing first group and 25 participants in the unappealing first group. Of the 50 participants, 24 (nine in the appealing first and 15 in the unappealing first groups) completed the experiment individually in a laboratory. The remaining 26 participants (16 in the appealing first and ten in the unappealing first groups) were tested on the online experiment building program Gorilla (gorilla.sc).

### Apparatus and materials

Trial presentation and recording of responses in the laboratory was controlled via PsyScope, Version 2.0 (Cohen et al., 1993). The stimuli were presented on a 19-in. screen connected to a Mac computer while online presentation was controlled via Gorilla.

**Fig. 2:**
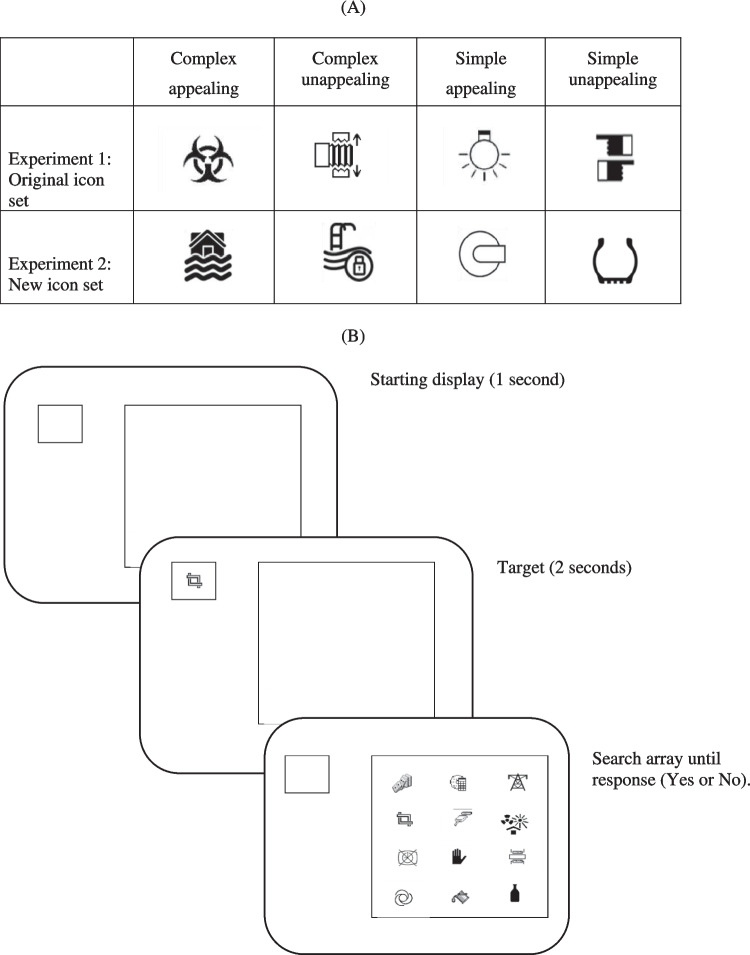
(**A**) Examples of target icons used in Experiment 1 (top row) and Experiment 2 (bottom row). (**B**) Examples of a target present trial

Icons for the visual search task were selected from a corpus of 239 icons where icon ratings had been obtained to measure visual appeal (McDougall & Reppa, 2008), visual complexity, concreteness and familiarity (McDougall et al., 1999). A total of 40 icons were chosen to use as target icons, with ten icons for each type (complex appealing, simple appealing, complex unappealing, simple unappealing). Example icons appear in Fig. 2.

**Fig. 3:**
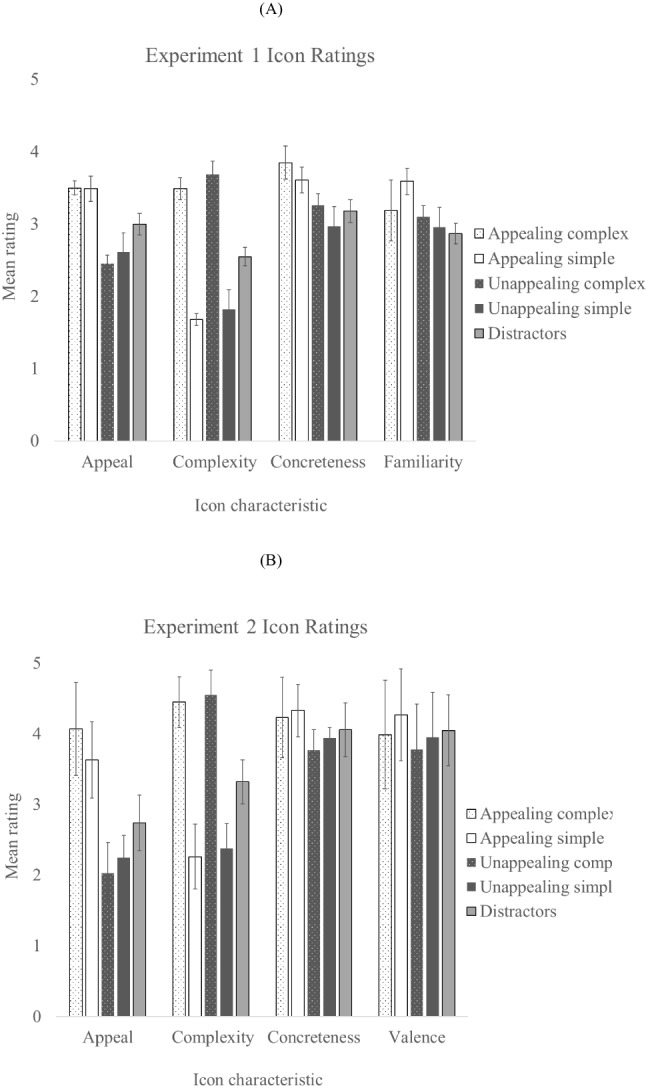
Mean ratings for each icon type on four icon characteristics – appeal, visual complexity, concreteness and familiarity, for icons used in Experiment 1 (**A**) and in Experiment 2 (**B**)

Seventy-two icons from the original icon corpus were used as distractors. They were selected to be neutral (mid-range) in terms of appeal and visual complexity The mean ratings for each icon type on the four icon characteristics (appeal, visual complexity, concreteness, and familiarity) appear in Fig. 3.

Univariate ANOVAs followed by Newman Keuls comparisons were carried out to examine differences between each type of icon for each icon characteristic. These showed that appealing complex and simple icons were significantly more appealing than unappealing complex and simple icons, *F*(4,110) =16.08, *p* < .001. Newman Keuls comparisons showed no difference in appeal ratings between appealing complex and appealing simple icons (*p* > .05) or between unappealing complex and simple icons (*p* > .05). Appeal ratings for distractor icons were significantly lower than appealing icons and significantly higher than unappealing icons (AC>D, AS>D, UC<D, US<D). Similarly, complex appealing and unappealing icons were significantly rated as more visually complex than simple appealing and unappealing icons, *F*(4, 110) =16.08, *p* < .001. Complex icons were rated as more complex than the distractor icons, who in turn were more complex than the visually simple icons (AC>D, AS<D, UC>D, US<D). Importantly, icon concreteness and familiarity were controlled and did not differ between icon types, *F*(4,110) =1.65, *p* > .05, and *F*(4,110) =1.94, *p* > .05, respectively.

Mood and arousal were measured using the self-assessment manikin (SAM) scale (e.g., Bradley & Lang, 1994). SAM is a visual analogue scale that measures valence, arousal and dominance on a 9-point Likert scale.

#### Design

##### Response times (RTs)

The visual search task was based on 2 (Target Presence: present vs. absent) × 2 (Target Appeal: appealing vs. unappealing) × 2 (Visual Complexity: complex vs. simple) × 4 (Set Size: 3 vs. 6 vs. 9 vs. 12) × 2 (Presentation Order: appealing first vs. unappealing first) mixed design, with Presentation Order as the between-participants variable. Twenty-six participants did the appealing target block first, followed by the unappealing target block, and the order was reversed for remaining 25 participants. In each block, there were 160 trials, yielding a total of 320 trials per participant. The dependent measure was RT in milliseconds.

##### Mood and arousal

Mood and arousal were measured at three time points for both Presentation Order participant groups: at baseline before the experiment, after block 1, and after block 2.

#### Procedure

Participants first completed the SAM mood measurement scale, before proceeding to do the first visual search block (see Fig. 1B for trial illustration). Each trial of the visual search task began with a screen with empty placeholders presented for 1 s. Next, a target icon appeared in in the top left corner of the screen for 2 s. After the target icon disappeared, three, six, nine or 12 icons appeared in random locations in the right part of the screen. The array either contained only distractor icons (in the target-absent conditions) or the target icon along with distractor icons (in the target-present conditions). The target icon was present in half of the trials in each block of trials. Participants decided whether the target icon was present in the array or not by pressing either ‘M’ (present) or ‘Z’ (not present) keys on the keyboard. In the lab-based experiment, incorrect responses were indicated via a beeping sound (500 ms). In the online version of the experiment, to ensure feedback was received even in the absence of audio capabilities of individual computers, images of a red cross or green tick appeared briefly (500 ms) on a screen to indicate correct/incorrect responses, and participants progressed to the next trial.

### Results and discussion

#### RT analyses

Trials with error responses were excluded (6.46%). Trials with RTs that were 3 standard deviations above or below the mean per participant per condition (0.35%) were removed from the correct RT analysis and not analysed further. A 2 (Appeal: appealing vs. unappealing) × 2 (Complexity: complex vs. simple) × 4 (Set size: 3, 6, 9 and 12) × 2 (Presentation Order: appealing first, unappealing second vs. unappealing first, appealing second) mixed ANOVA was carried out on *target-present* correct RTs with Presentation Order manipulated between participants. Significant interactions were examined with Bonferroni-Holm-corrected pairwise comparisons, and Cohen’s* d* is reported for all pairwise comparisons. To facilitate communication of the most relevant findings, we present each result in the context of the study’s research questions, i.e., those relating to (a) the role of appeal in visual search, (b) the effects of task experience and task switching, and (c) the efficiency of visual search as measured by examining the effects of set size. The results are shown and findings summarised in Tables 1 and 2 for target-present and target-absent trials, respectively. Cell means are illustrated in Online Supplementary Fig. 1.

**Table 1:** Experiment 1: Target-Present analysis

ANOVA main effects and interactions	F, p and ${\eta }_{p}^{2}$ values Cohen’s d for pairwise comparisons	Summary of findings
*The role of appeal in visual search*		
Appeal	*F*(1,48) = 9.27, *p* < .01, ${\eta }_{p}^{2}$ = .16	Search faster for appealing than for unappealing icons
Complexity	*F*(1,48) = 165.80, *p* < .001, ${\eta }_{p}^{2}$ = .77	Search faster for simple than for complex icons
Appeal × Complexity	*F*(1,48) = 8.09, *p* < .01, ${\eta }_{p}^{2}$= .14	Search times for complex icons but not for simple icons were affected by icon appeal
	Simple icons comparison: *t*(48) = 1.10, *p* = .27	Simple appealing and simple unappealing icons were found equally fast
	Complex icons comparison: *t*(48) = 4.09, *p* < .001, *d* = .58	Complex appealing icons found faster than complex unappealing icons
*Task experience and task switching*		
Presentation Order	*F*(1,48) = 5.23,* p* = .03, ${\eta }_{p}^{2}$= .10	Significant effect of Presentation Order on search times
Presentation Order × Appeal	*F*(1,48) = 6.38, *p* = .02, ${\eta }_{p}^{2}$= .12	Search times for appealing and unappealing stimuli depended on whether they appeared in the first or the second block of trials
	Appealing vs. unappealing icon comparison for *unappealing first/appealing second* group: *t*(24) = 6.16, *p* < .001, *d* = 1.24	Experience with the search task in block 1, led to faster search times for appealing icons in block 2
	Appealing vs. unappealing icon comparison for the *appealing first/unappealing second* group:*t*(24) = 1.38, *p* = 0.18	Despite participants having had experience with the search task in block 1, they did not benefit from that practice when they had to search for unappealing icons in block 2
*Efficiency of visual search: The effects of set size*		
Set Size	*F*(3,144) = 39.67, *p* < .001, ${\eta }_{p}^{2}$ = .45	Search times increased as set size increased
Set Size × Appeal	*F*(3,144) = 1.97, *p = .12*	Search times for appealing and unappealing icons were similarly affected across Presentation Order groups
Set Size × Complexity	*F*(3,144) = 5.90, *p* < .001, ${\eta }_{p}^{2}$ = .11	Search times for complex icons affected more by increasing set size resulting in steeper search slopes for complex icons (22 ms/item), compared to simple icons (15 ms/item)

Outcome of 2 (Appeal: appealing vs. unappealing) × 2 (Complexity: complex vs. simple) × 4 (Set Size: 3, 6, 9, 12) × 2 (Presentation order: appealing first, unappealing second vs. unappealing first, appealing second) mixed analysis of variance with Appeal, Complexity and Set Size as repeated measures.^1^ Results of this analysis are illustrated in Supplementary Fig. 1A

^1^Results are shown for the role of appeal in visual search, the effects of task experience and switching, and the effect of set size in turn

**Table 2:** Experiment 1: Target-Absent analysis

ANOVA main effects and interactions	F, p and ${\eta }_{p}^{2}$ values	Summary of findings
	Cohen’s d for pairwise comparisons	
*The role of appeal in visual search*		
Appeal	*F*(1,48) = 9.27, *p < .01,* ${\eta }_{p}^{2}$ = .16	Search faster for appealing than for unappealing icons.
Complexity	*F*(1,48) = 54.32, *p* < .001, ${\eta }_{p}^{2}$ = .53	Search faster simple than for complex icons.
Appeal × Complexity	*F*(1,48) = 23.50; *p* < .001,${\eta }_{p}^{2}$ = .32	Search times for complex icons were affected by icon appeal but not simple icons.
	Simple icons comparison: *t*(48) = 1.12, *p* = .27	Simple appealing and simple unappealing icons were found equally fast.
	Complex icons comparison: *t*(48) = 5.74, *p* < .001, *d* = .41	Complex appealing icons found faster than complex unappealing icons.
*Task experience and task switching*		
Presentation order	*F*(1,48) = 1.94, *p* = .17	No overall effect of Presentation Order on search times.
Presentation order × Appeal	*F*(1,48) = 22.04, *p* < .001, ${\eta }_{p}^{2}$ = .31	Search times for appealing and unappealing stimuli depended on whether they appeared in the first or the second block of trials.
	Appealing vs. unappealing icon comparison for *unappealing first/appealing second* group: *t*(24) = 6.19, *p* < .001, *d* = .55	Experience with the search task in Block 1, led to faster search times for appealing icons in Block 2.
	Appealing vs. unappealing icon comparison for the *appealing first/unappealing second* group: *t*(24) = .47, *p* = 1.00	Despite participants having had experience with the search task in Block 1, they did not benefit from that practice when they had to search for unappealing icons in Block 2.
*Efficiency of visual search: The effects of set size*		
Set Size	*F*(3,144) = 198.30, *p* < .001, ${\eta }_{p}^{2}$ = .80	Search times increased as set size increased.
Set Size × Appeal	*F*(3,144) = 2.35, *p <* .01, ${\eta }_{p}^{2}$ = .10	Search termination times for appealing and unappealing icons were similarly affected across Presentation Order groups.
Set Size × Complexity	*F*(3,144) = 8.94; *p* < .001, ${\eta }_{p}^{2}$ = .16	Search termination slopes were steeper for complex than for simple icons.
Set Size × Appeal × Complexity	*F*(3,144) = 8.94; *p* < .001, ${\eta }_{p}^{2}$ = .16	Search termination slopes were affected by icon appeal for complex icons but not for simple icons
	Simple icons comparison: *t*(48) = .28, *p* = .78	No difference in search termination slopes between simple appealing (35 ms/item) and simple unappealing (38 ms/item) icons.
	Complex icons comparison:* t*(48) = 2.98, *p* < .01, *d* = .42	Steeper search termination slopes for complex unappealing (45 ms/item).

Outcome of 2 (Appeal: appealing vs. unappealing) × 2 (Complexity: complex vs. simple) × 4 (Set Size: 3, 6, 9, 12) × 2 (Presentation order: appealing first, unappealing second vs. unappealing first, appealing second) mixed analysis of variance with Appeal, Complexity and Set Size as repeated measures. Results of this analysis are illustrated in Supplementary Fig. 1B

#### Target-present RT analyses

##### Effect of appeal in visual search for icons varying in appeal and complexity

There was a significant main effect of *Appeal* on search times and the *Appeal × Complexity* interaction was significant. Pairwise comparisons showed simple appealing and unappealing icons were found equally fast; however, complex appealing icons were found faster than complex unappealing icons.

##### Task experience and task switching

**Fig. 4:**
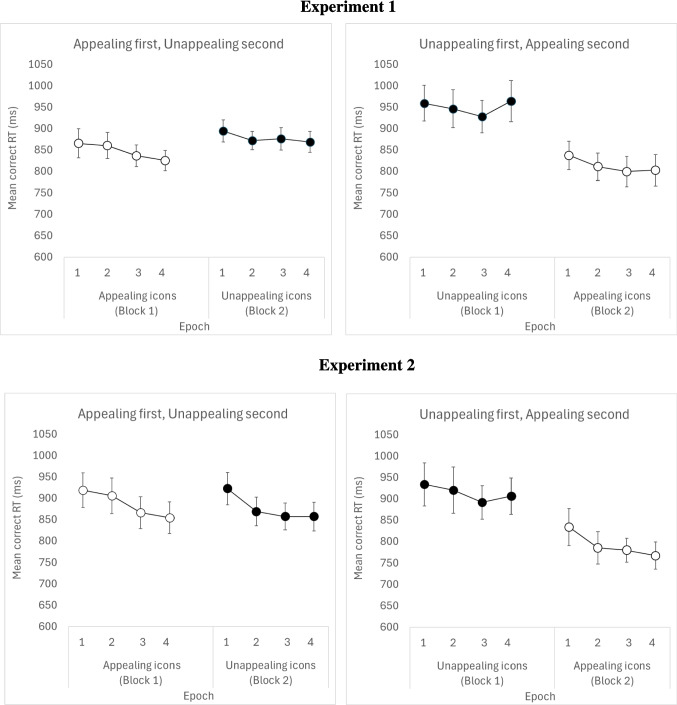
Presentation Order by Appeal interaction in Experiment 1 and Experiment 2. The difference between the two experiments was the stimuli used (see Methods for details). The same pattern of results is revealed in both experiments. In appealing first / unappealing second group, appealing icons appeared in Block 1, and unappealing icons appeared in Block 2. The opposite order was followed for the unappealing first / appealing second group. The mean RT includes both target present and target absent trials. Epochs represent 25% of the trials in each block– that is, each epoch contains 40 trials per participant

The main effect of Presentation Order was not significant but was qualified by a significant Presentation Order × Appeal interaction, illustrated in Fig. 4. In the *appealing first/unappealing second* group, there was no difference in search times for search times between appealing and unappealing items. This suggests that experience with search task in block 1 did not benefit search times in block 2 when searching for unappealing icons. However, in the unappealing first/appealing second group, *search was faster for appealing* compared to unappealing items. This suggests that experience with the search task in block 1 benefitted search times for appealing icons in block 2. There were no other significant interactions.

##### Efficiency of visual search: The effects of set size

Search slopes significantly increased with slower search response times as set size increased. Search times for complex icons were affected more by set size increased resulting in a significant interaction between set size and complexity, suggesting more efficient searches for simple compared to complex icons. Search times for appealing icons were faster than for unappealing icons, and search for either appealing or unappealing icons was not affected by set size. Therefore, overall, search for appealing icons was not necessarily more efficient than for unappealing icons.

#### Target-absent RT analysis

An identical ANOVA analysis was carried out for target absent trials. Results are summarised in Table 2 and illustrated in Supplementary Fig. 1. As can be seen from Table 4, the effects observed in the target absent analysis closely mirrored those found in the target-present analysis.

#### The effect of search task experience and appeal on search performance

To investigate how practice interacts with stimulus appeal, we divided each block’s RTs into *epochs*, with each epoch comprising 25% of each block’s trials. The analysis is based on the average RT collapsed across visual complexity and set size, as neither variable had interacted with block in the earlier analysis. Because the pattern of search RT was similar for target-present and target-absent trials, the analysis was based on the average RT of target-present and target-absent trials. Means per condition are shown in Fig. 4.

A 2 (Appeal: appealing vs. unappealing) × 4 (Epoch: 1, 2, 3 and 4) × Presentation Order (appealing first – unappealing second vs. unappealing first – appealing second) mixed ANOVA with Presentation Order as the between-participants measure, on correct mean RT revealed a significant main effect of Appeal,* F*(1,47) = 20.22, *p* < .001, ${\eta }_{p}^{2}$ = .31, with appealing icons yielding shorter search times than unappealing icons. There was a significant effect of Epoch, *F*(3,141) = 3.03, *p* = .03, ,${\eta }_{p}^{2}$= .07, with search RT generally decreasing over epochs – as task experience increased within each block. The main effect of Presentation Order was not significant, *F*(1,47) = .48, *p* = .49, but it was involved in a significant Appeal × Presentation Order interaction, *F*(1,47) = 8.40, *p* = .006,${\eta }_{p}^{2}$= .15, illustrated in Fig. 4. This underscores the findings from the previous analysis showing clearly that appealing icons benefit from prior task experience when presented in block 2 but this is not the case for unappealing icons with RTs remaining at similar levels for both block 1 and block 2. There were no other significant interactions.

To specifically examine if the practice effect was influenced by the intrinsic appeal of the stimuli, we analysed the magnitude of RT reductions across epochs within each stimulus condition.^1^ We collapsed across presentation order and examined the decrease of RT across the four epochs for each stimulus appeal condition. Simple effects analyses showed that for appealing stimuli (regardless of whether they appeared first or second block) there was a significant decrease in RT from epoch 2 to 3 (*p* < .05), while the decrease in RT between epochs 1 and 2 and 3 to 4 were not significant (*p* > .05). For unappealing icons, there were no significant decreases in RT between any of the epochs (*p* > .05).

#### Mood and arousal analyses

**Fig. 5.**
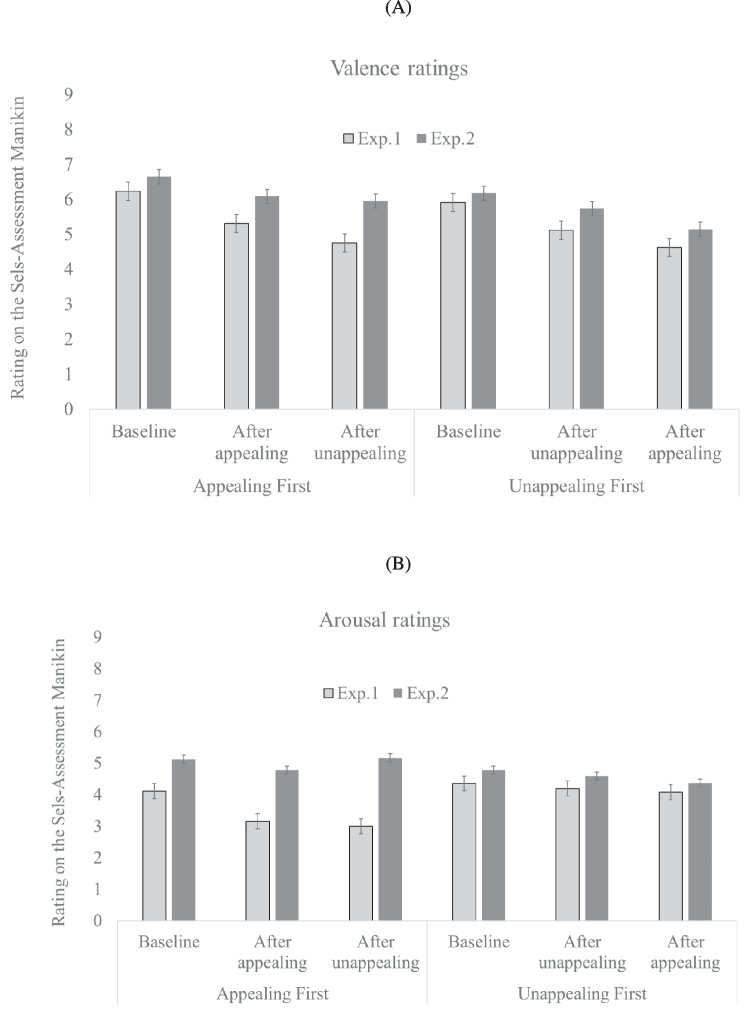
Mean valence (**A**) and arousal (**B**) ratings at three different times points for Experiment 1 and Experiment 2

Mean valence and arousal scores on the self-assessment manikin are shown in Fig. 5. A mixed 3 (Time: at baseline, after block 1, after block 2) × 2 (Presentation Order: appealing first vs. unappealing first) ANOVA on *valence* scores revealed a significant main effect of Time, *F*(2,94) = 28.00, *p* < .001; ${\eta }_{p}^{2}$= .373, with valence becoming lower from baseline to after block 2. The main effect of Presentation Order was not significant, *F*(1,47) = .29, *p* = .59, and neither was the interaction *F*(2,94) = .13, *p* = .88.

A mixed 3 (Time: baseline, after block 1, after block 2) × 2 (Presentation Order: appealing first vs. unappealing first) ANOVA on *arousal* scores showed a significant main effect of Time, *F*(2,94) = 3.41; *p* = .04, ${\eta }_{p}^{2}$ = .07. The main effect of Presentation Order was not significant, *F*(1,47) = 3.06, *p* = .09, and neither was the Time × Presentation Order interaction, *F*(2,94) = 3.79; *p* = .07.

To summarise, the findings from Experiment 1 showed that both visual complexity and visual appeal influenced search performance with faster searches for simple over complex items, and for appealing over unappealing items. Second, as we have found previously, visual complexity of the target influences search efficiency. Third, having task experience did not transfer to more efficient performance in later trials that used unappealing icons. In contrast, having experience with the task led to even more efficient search performance in later trials that use appealing icons. Finally, mood and arousal were not significantly influenced by the appeal value of target icons. Those findings are discussed in detail in the General discussion.

## Experiment 2: The effects of appeal on visual search using new icons

Experiment 2 replicated Experiment 1 using a novel contemporary set of icons. This was important to ensure that our findings generalised to other icons sets, particularly those using more up to date design principles (e.g., Brysbaert, 2007; Collaud et al., 2022). As before, participants carried out a visual search task, seeing either appealing targets in the first block and unappealing targets in the second block or vice versa.

### Participants

Fifty participants were recruited (40 female, ten male, age 19–75 years), with reported normal or corrected-to-normal vision. Their mean age was 33.42 years (*SD* = 12.92). We recruited 23 participants in the appealing first group and 27 participants in the unappealing first group. Participants received either four participant pool credits or £8.50 for approximately 45 min of their time.

#### Apparatus and materials

Trial presentation and recording of responses was controlled via the online experiment building program Gorilla (gorilla.sc).

Icons for the visual search task were selected from a corpus of 600 icons reported by McDougall et al. (2023). In that corpus, there were not familiarity ratings, so we used icons that were instead matched in terms of valence (i.e., the pleasantness or unpleasantness of the icon). A total of 40 icons were chosen to use as target icons, with ten icons for each type (complex appealing, simple appealing, complex unappealing, simple unappealing). Example icons appear in Fig. 2. Seventy-two new icons were chosen to be used as distractors. The mean ratings for each target icon type and the distractor icons for icon characteristics (appeal, visual complexity, concreteness, and valence) appear in Fig. 3. Distractors were chosen to be neutral in terms of rated appeal and visual complexity, and to not differ from the target icons in terms of concreteness and valence.

A set of univariate ANOVAs showed differences between the Icon Types in terms of rated visual complexity and appeal, and the lack of difference in terms of concreteness and valence.

As in Experiment 1, we carried out a set of univariate ANOVAs followed by Newman Keuls comparisons were carried out to examine differences between each type of icon for each icon characteristic. These showed that appealing complex and simple icons were significantly more appealing than unappealing complex and simple icons *F*(4, 110) = 42.35, *p* < .001. Newman-Keuls comparisons showed no difference in appeal ratings between appealing complex and appealing simple icons (p > .05) or between unappealing complex and simple icons (p > .05). Appeal ratings for distractor icons were significantly lower than appealing icons and significantly higher than unappealing icons (AC>D, AS>D, UC<D, US<D).

In terms of visual complexity ratings, complex appealing and unappealing icons were significantly rated as more visually complex than simple appealing and unappealing icons, *F*(4,110) = 14.48, *p* < .001. Newman-Keuls comparisons showed that complex icons were rated as more complex than the distractor icons, who in turn were more complex than the visually simple icons (AC>D, AS<D, UC>D, US<D). Importantly, icon concreteness and familiarity were controlled and did not differ between icon types, *F*(4,110) = .26, *p* = .92, and *F*(4, 110) = 0.47, *p* > .05, respectively.

#### Design and procedure

Design and procedure were identical to Experiment 1.

### Results and discussion

The results of Experiment 2 replicate the main findings of Experiment 1.

#### RT analyses

Trials with error responses were excluded (5.49%). Trials with RTs that were 3 standard deviations above or below the mean per participant per condition (0.28%) were removed from the correct RT analysis and not analysed further. Cell means appear in Supplementary Fig. 2. A 2 (Appeal: appealing vs. unappealing) × 2 (Complexity: complex vs. simple) × 4 (Set size: 3, 6, 9 and 12) × 2 (Presentation Order: appealing first vs. unappealing first) mixed ANOVA was carried out on *target-present* and *target-absent* correct RTs with Presentation Order as between-participants factor. Significant interactions were examined with Bonferroni-Holm-corrected pairwise comparisons. Cohen’s* d* is reported for all pairwise comparisons. As in Experiment 1, results are presented in relation to the research questions. Search times are illustrated in Fig. 5 and the results of the statistical analyses are presented in Tables 3 and 4 for target-present and target-absent trials respectively.

**Table 3:** Experiment 2: Target-Present analysis

ANOVA main effects and interactions	F, p and ${\eta }_{p}^{2}$ values	Summary of findings
	Cohen’s *d* for pairwise comparisons	
*The role of appeal in visual search*		
Appeal	*F*(1,48) = 10.44, *p* < .01, ${\eta }_{p}^{2}$ = .18	Search faster for appealing than for unappealing icons
Complexity	*F*(1,48) = 64.12, *p* < .001, ${\eta }_{p}^{2}$ = .58	Search faster simple than for complex icons
Appeal × Complexity	*F*(1,48) = .21, *p* = .65	Search time for all icons were affected by icon appeal regardless of visual complexity
*Task experience and task switching*		
Presentation Order	*F*(1,48) = .41,* p* = .52	No overall effect of Presentation Order on search times
Presentation Order × Appeal	*F*(1,48) = 30.02, *p* < .001, ${\eta }_{p}^{2}$ = .37	Search times for appealing and unappealing stimuli depended on whether they appeared in the first or the second block of trials
	Appealing vs. unappealing icon comparison for *unappealing first/appealing second* group: *t*(24) = 4.88, *p* < .001, *d* = .94	Experience with the search task in block 1, led to faster search times for appealing icons in block 2
	Appealing vs. unappealing icon comparison for the *appealing first/unappealing second* group: *t*(24) = 1.51, *p* = .09.	Despite participants having had experience with the search task in block 1, they did not benefit from that practice when they had to search for unappealing icons in block 2
*Efficiency of visual search: The effects of set size*		
Set Size	*F*(3,144) = 119.66, *p* < .001, ${\eta }_{p}^{2}$ = .72	Search times increased as set size increased
Set Size × Appeal	*F*(3,144) = 1.66, *p =* .41	Search times for appealing and unappealing icons were similarly affected across Presentation Order groups
Set Size × Complexity	*F*(3,144)=.76, *p*=.52.	Search times were similar for complex and simple icons as set size increased

Outcome of 2 (Appeal: appealing vs. unappealing) × 2 (Complexity: complex vs. simple) × 4 (Set Size: 3, 6, 9, 12) × 2 (Presentation order: appealing first, unappealing second vs. unappealing first, appealing second) mixed analysis of variance with Appeal, Complexity and Set Size as repeated measures. Results of this analysis are shown in Supplementary Fig. 2A

**Table 4:** Experiment 2: Target Absent analysis

ANOVA main effects and interactions	F, p and ${\eta }_{p}^{2}$ values	Summary of findings
	Cohen’s d for pairwise comparisons	
*The role of appeal in visual search*		
Appeal	*F*(1,48) = .51, *p* = .48	No effect of appeal on search times
Complexity	*F*(1,48) = 1.73, *p* = .19	No effect of visual complexity on search times
Appeal × Complexity	*F*(1,48) = 1.37; *p* = .25	No interaction between appeal and visual complexity
*Task experience and task switching*		
Presentation Order	*F*(1,48) = 1.01, *p* = .37	No overall effect of Presentation Order on search times
Presentation Order × Appeal	*F*(1,48) = 3.97, *p* < .02, ${\eta }_{p}^{2}$ = .08	Search times for appealing and unappealing stimuli depended on whether they appeared in the first or the second block of trials
	Appealing vs. unappealing icon comparison for *unappealing first/appealing second* group: *t*(26) = 6.60, *p* < .001, *d* = 1.27.55	Experience with the search task in block 1, led to faster search times for appealing icons in block 2
	Appealing vs. unappealing icon comparison for the *appealing first/unappealing second* group: *t*(22) = 1.31, *p* = 2.00	Despite participants having had experience with the search task in block 1, they did not benefit from that practice when they had to search for unappealing icons in block 2
*Efficiency of visual search: The effects of set size*		
Set Size	*F*(3,144) = 233.56, *p* < .001, ${\eta }_{p}^{2}$ = .82	Search times increased as set size increased
Set Size × Appeal	*F*(3,144) = 1.53; *p* = .21	No effect of appeal on search termination slopes
Set Size × Complexity	*F*(3,144) = .38; *p* = .76	No effect of visual complexity on search termination slopes
Set Size × Appeal × Complexity	*F*(3,144) = 3.20; *p =* .2, ${\eta }_{p}^{2}$ = .06	Search termination slopes were affected *to some extent* by icon appeal for complex icons but not for simple icons
	Simple icons comparison: *t*(48) = 1.42, *p* = .32, *d* = .10	No difference in search termination slopes between simple appealing (41 ms/item) and simple unappealing (40 ms/item) icons
	Complex icons comparison:* t*(48) = 3.19, *p* < .001, *d* = .39	Steeper search termination slopes for complex unappealing (55 ms/item) compared to complex appealing icons (45 ms/item)

Outcome of 2 (Appeal: appealing vs. unappealing) × 2 (Complexity: complex vs. simple) × 4 (Set Size: 3, 6, 9, 12) × 2 (Presentation order: appealing first, unappealing second vs. unappealing first, appealing second) mixed analysis of variance with Appeal, Complexity and Set Size as repeated measures. Results of this analysis are illustrated in Supplementary Fig. 2B

#### Target-present RT analyses

##### Effect of appeal in visual search for icons varying in appeal and complexity

The main effect of Appeal was significant with appealing icons found faster than unappealing icons (see Table 3). There was also a strong effect of icon Complexity with simple icons found faster than complex icons. There was no interaction between icon appeal and complexity, but the two were involved in a three-way interaction with Presentation Order (see below).

##### Task experience and task switching

As in Experiment 1, the main effect of Presentation Order was not significant but the interaction between Presentation Order and Appeal was significant. Pairwise comparisons again revealed that in the unappealing first-appealing second group, experience with the search task in block 1 led to faster search times for appealing icons in block 2. However, in the *appealing first-unappealing second* group, experience with the search task showed no benefit when unappealing icons were presented in block 2.

##### Efficiency of visual search: The effects of set size

The main effect of *Set Size* was significant with slower RT in larger arrays (see Supplementary Fig. 2). In contrast to Experiment 1, there was no interaction between set size and visual complexity.

The results of the target-present RT analysis effectively replicate those in Experiment 1.

#### Target-absent RT analyses

##### Effect of appeal in visual search for icons varying in appeal and complexity

Neither icon appeal nor icon complexity had significant effects on search termination times, but they were involved in a significant Appeal × Complexity × Set Size interaction (see Table 4).

##### Efficiency of visual search: The effects of set size

The main effect of Set Size was significant with search times becoming slower as search array sizes increased (Supplementary Fig. 2). *Complex appealing* icons had flatter slopes than *complex unappealing* icons, while there was no difference in slopes between *simple appealing and simple unappealing* icons. This finding replicates previous work by showing that effects of appeal are evident when the task is harder – i.e., when looking for visually complex icons (e.g., Moshagen et al., 2009; Reppa & McDougall, 2015, 2022).

##### Task experience and task switching

The main effect of Presentation Order was not significant, but it was involved in an Appeal × Presentation Order interaction, illustrated in Fig. 4. There was no difference in termination times between appealing and unappealing items when appealing icons appeared in the first block of trials followed by unappealing icons in the second block did not benefit search termination in block 2. However, *search was terminated faster for appealing* compared to unappealing items when they appeared in block 2, following experience with the visual search task search in block 1. There were no other significant interactions. Again, the results replicate those of the target-absent RT analysis in Experiment 1.

##### The effect of search task experience and appeal on search times

As in Experiment 1, correct participant RTs were divided into epochs, with each epoch comprising 25% of the block’s trials (both target present and target absent). Cell means are shown in Fig. 4 (bottom panel). A 2 (Appeal: appealing vs. unappealing) × 4 (Epoch: 1, 2, 3 and 4) × Presentation Order (appealing first, unappealing second vs. unappealing first, appealing second) mixed ANOVA with Presentation Order as the between-participants measure on correct mean search RT revealed a significant main effect of Appeal, *F*(1,48) = 12.17, *p* < .001, ${\eta }_{p}^{2}$= .20, with appealing icons yielding shorter search times than unappealing icons. There was a significant effect of Epoch, *F*(3,144) = 60.61, *p* < .001, ${\eta }_{p}^{2}$ = .12, with search RT generally decreasing over epochs – as task experience increased. The main effect of Presentation Order was not significant, *F*(1,48) = .2.27, *p* = .14, but it was involved in a significant Appeal × Presentation Order interaction, *F*(1,48) = 17.16, *p* < .001, ${\eta }_{p}^{2}$= .26, illustrated in Fig. 5. Pairwise comparisons within each group showed that for the appealing first group, there was no difference in search RT between appealing (which appeared in block 1) and unappealing icons (which appeared in block 2). However, for the unappealing first group, search RT for appealing icons (which appeared in block 2) was faster than for unappealing icons (which appeared in block 1). There were no other significant interactions.

As we did in Experiment 1, we analysed the magnitude of RT reductions across epochs within each stimulus appeal condition. Simple effects analyses showed that for appealing stimuli (collapsed across both blocks) there was a significant decrease in RT from epoch 2 to 3 (*p* < .05), while the decrease in RT between epochs 1 and 2 and 3 to 4 were not significant (*p* > .05). For unappealing icons, however, there were no significant decreases in RT between any of the epochs (*p* > .05). Those results further show that practice benefited appealing but not unappealing icons.

#### Mood and arousal analyses

Mean mood valence and arousal scores on the self-assessment manikin are shown in Fig. 5. A mixed 3 (Time: at baseline, after block 1, after block 2) × 2 (Presentation Order: appealing first vs. unappealing first) ANOVA on *valence* scores revealed a significant main effect of Time, *F*(2,96) = 10.53, *p* < .001;${\eta }_{partial }^{2}$= .18, with valence becoming increasingly lower from baseline to after block 2. The main effect of Presentation Order was not significant, *F*(1,48) = 1.71, *p* = .20, and neither was the interaction *F*(2,96) = .080; *p* = .45.

A mixed 3 (Time: baseline, after block 1, after block 2) × 2 (Presentation Order: appealing first vs. unappealing first) ANOVA on *arousal* scores showed no significant main effect of Time, *F*(2,96) = .51; *p* = .60. The main effect of Presentation Order was not significant, *F*(1,48) = 1.48; *p* = .23. neither was the interaction, *F*(2,96) = .70; *p* = .50.

## General discussion

Experiment 1 examined the effect of aesthetic appeal of visual stimuli (icons and symbols) on performance and its effect of appeal on mood and arousal. Three key findings were revealed. First, with respect to search performance overall, search times were faster when searching for appealing compared to unappealing icons. Second, with respect to mood and arousal, appeal did not influence self-reported mood and arousal scores– participants’ mood and arousal did not differ as a function of whether they dealt with appealing or unappealing icons. Finally, practice benefitted task performance mostly for appealing but not for unappealing icons. Each finding is discussed in turn.

### Visual appeal reduced search times

As in our earlier work (e.g., Reppa & McDougall, 2015, 2022; Reppa et al., 2021), when the target was present, appealing targets were found faster. When the target was absent, it took longer to terminate the search for unappealing targets, and there were steeper termination slopes when looking for complex unappealing targets. The two findings – that appealing targets were found faster, and that searches for appealing targets were terminated quicker suggests that appealing targets were processed faster than unappealing targets, especially when the targets were difficult to process to begin with, i.e., when they were visually complex.

### No discernible effect of appeal on mood and arousal

The design of the current study allowed to examine whether dealing with aesthetically pleasing stimuli might improve mood valence or increase arousal. Unlike earlier findings (e.g., Bhandari et al., 2019; Seo et al., 2015, 2016; Sonderegger et al., 2012), neither mood valence nor arousal were affected by aesthetic appeal. This may be because appeal does not influence mood, or because the current methodology did not allow such effects to be revealed. It could be that icons evoked emotional responses, but they were not strong enough to influence general mood. For instance, although the appeal of the target was manipulated, the distractor stimuli were neutral in terms of appeal. Therefore, any effect of target appeal may have been moderated by the presence of neutral distractors. Evidence from our laboratory has shown that distractor appeal interacts with target appeal to influence search times in a classic visual search task (e.g., Reppa & McDougall, 2022). Therefore, it is more than likely that any effects of target appeal on mood may be moderated by the presence of aesthetically neutral distractors. Alternatively, mood changes could be too subtle, and the measure we used did not capture it. Therefore, although no effect of appeal on mood was revealed here, it remains possible that appealing stimuli can influence mood, and future studies should examine this. It could also be that mood is only affected in subjective reports where a more conscious, reflective type of aesthetic appreciation is part of the task requirement (e.g., aesthetic responses to art or music) rather than subliminal appeal responses occurring automatically for all stimuli.

### Practice makes perfect but for appealing items

In the current study practice benefitted task performance for appealing but not for unappealing icons. Two lines of evidence support this conclusion. First, participants benefited from practice within the block of trials, but only when the icons were appealing. When looking for unappealing icons, there was no significant decrease of search times from the start to the end of the block of trials. Second, participants who did 160 trials of the visual search task, looking for *unappealing* icons were significantly faster to search for *appealing* icons in the second block of trials. However, no such benefit of practice was shown for unappealing target icons. Participants who searched for *appealing* icons in the first block of 160 trials, although they continually improved across the first block, when it came to searching for *unappealing* icons in the second block of trials, their search times were no different from those in the first block. Instead, search times jumped to the same level as they were in the first few trials of the first block, when they had very little experience with the task. Therefore, having task experience with appealing icons does not transfer to more efficient performance in later trials that use unappealing icons.

This drop in performance following the switch from appealing to unappealing icons is a sort of task-switching cost found elsewhere (e.g., Alport et al., 1994; Hillstrom, 2000; Rogers & Monsell, 1995). Task-switching costs have historically been caused by a change in task (i.e., from a naming task to a counting task), and by the change in stimuli – for instance, an irrelevant stimulus dimension, such as its colour, shape and, in the current study, level of aesthetic appeal or lack thereof, can *capture* attention and lead to significant costs in performance (e.g., Rubin & Koch, 2006).

In the current study, there was no switch of task – all participants did two identically designed visual search blocks, one after the other. Instead, the switch – i.e., the change between the two blocks – was the stimuli used during the search task in each of the blocks. Appeal of the target icons was an irrelevant attribute and in principle should and could have been ignored. However, it affected performance in a bottom-up manner. Switching from appealing to unappealing icons led to (a) a cost in performance at the start of the switch, and (b) to lack of improvement in search performance for the remainder of the search for unappealing icons. In contrast, switching from looking for unappealing icons to looking for appealing icons (a) led to no cost in search performance, but instead to a ‘*switch benefit’*, and (b) search performance continued to improve as participants search for appealing icons. Therefore, appeal seems to have counteracted the negative effects of stimulus switching on performance.

One possible mechanism by which appeal may facilitate learning is via motivation. In a recent study we showed that appealing stimuli act like other rewarding stimuli, such as money or food, to influence performance (e.g., Reppa et al., 2021). Furthermore, evidence from neuroimaging studies has shown that aesthetic judgement is correlated with activity in neural systems underlying reward, for auditory (e.g., Blood & Zatorre, 2001) and visual stimuli (e.g., Aharon et al., 2001; Kawabata & Zeki, 2004; Kirk et al., 2009; Leder et al., 2004; Vartanian & Goel, 2004). Therefore, the beneficial effect of appeal on improving performance over time is likely to related to the potentially motivating nature of aesthetically pleasing stimuli. The current study corroborates the hypothesis that appealing objects can be rewarding, hence the continuous improvement in search performance across the experiment (see Fig. 4, the two rightmost columns). The continuous improvement in search performance for appealing stimuli is also compatible with the idea that with more exposures appealing stimuli, like faces (e.g., Han et al., 2020; Reppa at al., 2021) become more appealing over time. As stimuli increase in appeal, and therefore their value increases leading to better, more motivated performance.

That *practice makes perfect* more *for appealing* *stimuli* but not for unappealing stimuli is a novel finding with significant implications for learning. That appealing stimuli benefit more with practice than their unappealing counterparts speaks to its power to facilitate learning, a possibility for future examination.

## Supplementary information

Below is the link to the electronic supplementary material.

Supplementary file1 (DOCX 881 KB)

## Data Availability

The materials used and the data reported in this article can be obtained by contacting the corresponding author, Dr. Irene Reppa (i.reppa@swansea.ac.uk).

## Footnotes

1 We thank an anonymous reviewer for this suggestion.
