# Supplementary material for: Practice makes perfect, especially when doing what we like
Source: Atten Percept Psychophys. 2025 Feb 24;87(3):981–97. doi: 10.3758/s13414-025-03031-8 (PMC11965255; doi:10.3758/s13414-025-03031-8)
Supplement: Supplementary file 1 — Supplementary file1 (DOCX 881 KB) [file 13414_2025_3031_MOESM1_ESM.docx]

**Supplementary Figure 1**: Mean RT for icons varying in appeal and complexity in Experiment 1 for (A) target present and (B) target absent trials. Error bars indicate standard error of the mean.

(A)


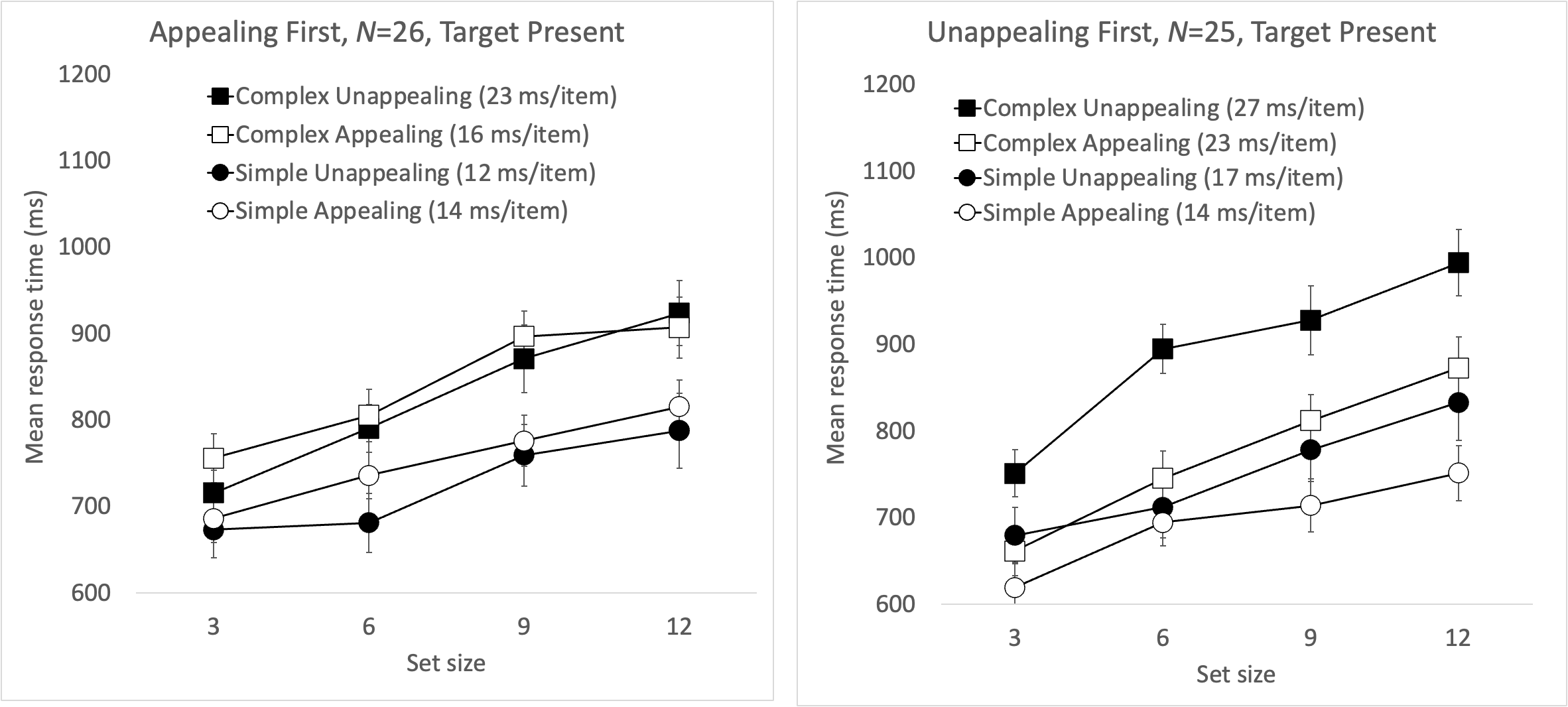


(B)


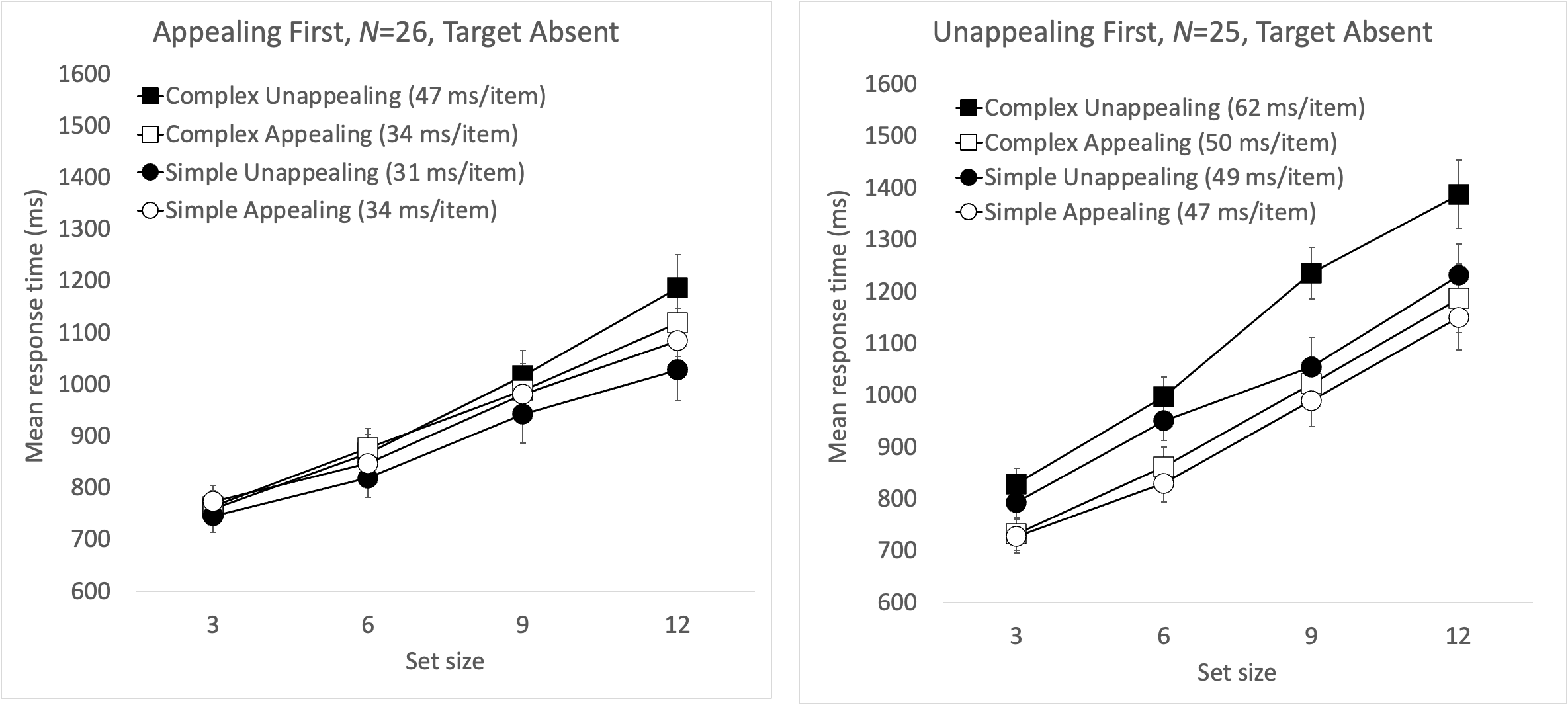


**Supplementary Figure 2:** Mean RT per condition in Experiment 2, for (A) target present and (B) target absent trials. Error bars indicate standard error of the mean.

(A)
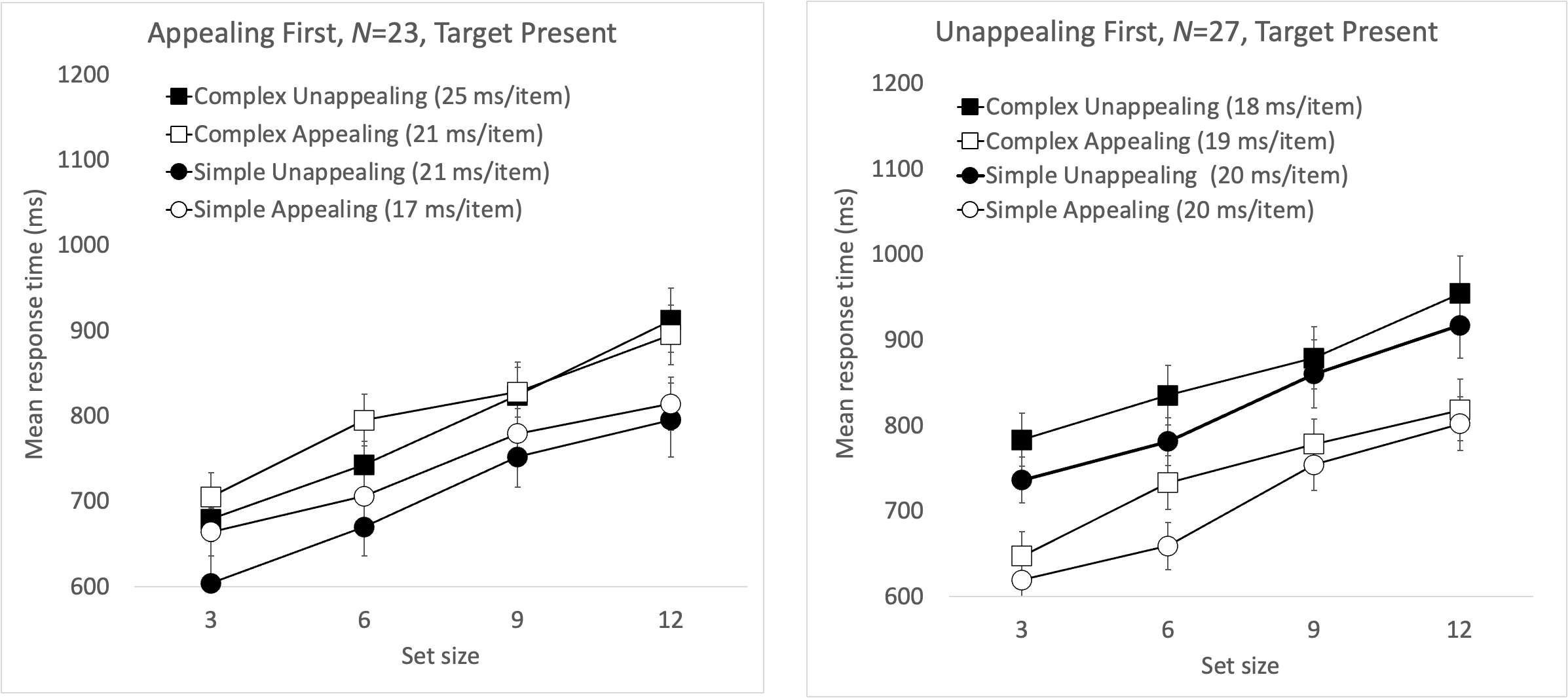


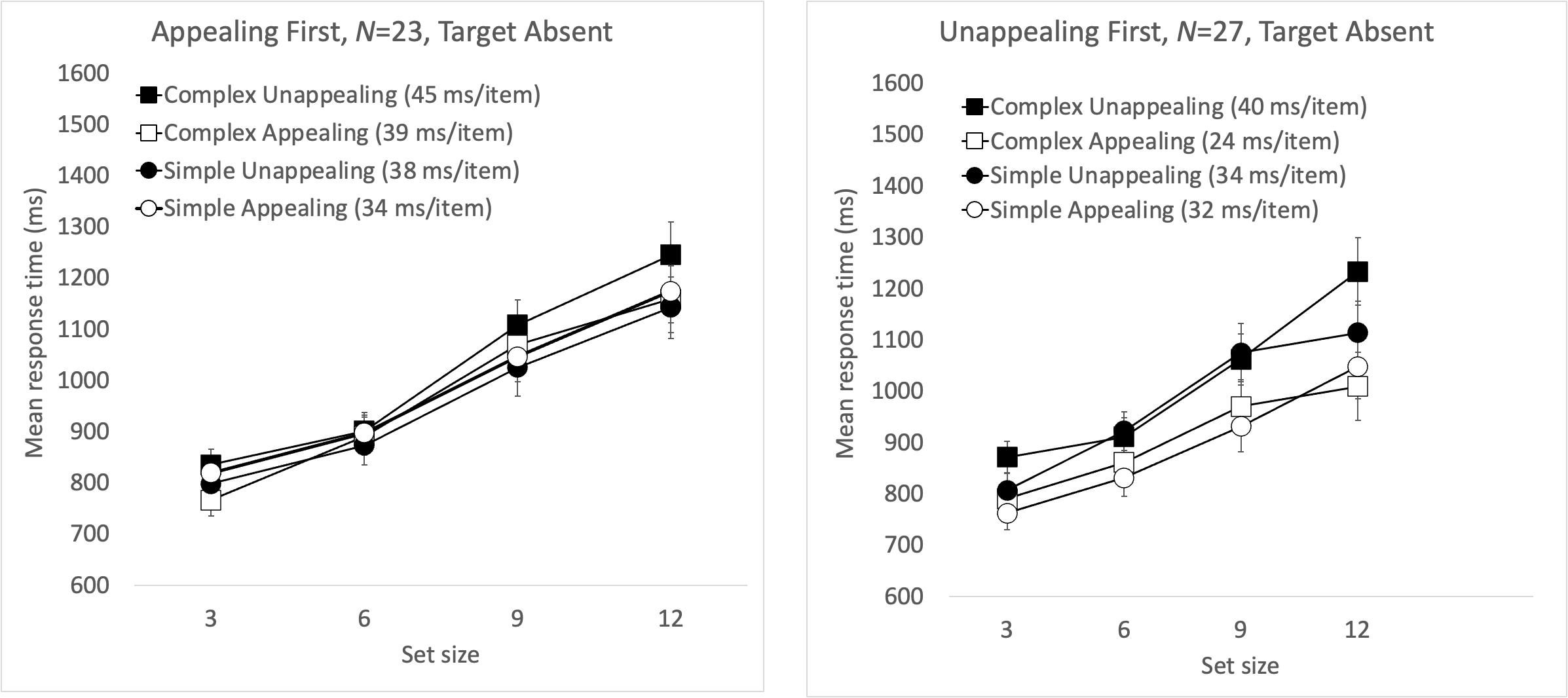
(B)
